# Tannic acid inhibits pain mediators, inflammation and oxidative stress in mice exposed to glyphosate-based herbicide

**DOI:** 10.5620/eaht.2024019

**Published:** 2024-06-21

**Authors:** Patrick Oluwole Abolarin, Bamidele Victor Owoyele

**Affiliations:** 1 Department of Physiology/Pharmacology, Chrisland University, College of Basic Medical Sciences, Abeokuta, Ogun state, Nigeria; 2 Department of Physiology, Neuroscience and Pain Laboratory, College of Health Sciences, University of Ilorin, Ilorin, Kwara state, Nigeria

**Keywords:** Glyphosate, tannic acid, pain perception, neuroinflammation, antioxidant enzymes

## Abstract

Chronic exposure to glyphosate-based herbicide (Gly) has been associated with neurological disorders. Tannic acid (TA) is an antioxidant with attenuating action against neuroinflammation-associated conditions. This study evaluated the effect of Gly on pain perception alongside antinociceptive and anti-inflammatory actions of TA in Gly-exposed mice. Male Swiss mice were randomly divided into six groups (n=8): control (distilled water 0.2 ml/kg), Gly (Gly 500 mg/kg), Pre-TA + Gly (TA 50 mg/kg pre-treatment, afterwards Gly-administered), TA + Gly (TA 50 mg/kg and Gly co-administered), Pre-AA + Gly (ascorbic acid (AA) 10 mg/kg pre-treatment, afterwards Gly-administered), and AA + Gly (AA 10 mg/kg and Gly co-administered). Mechanical, thermal, and chemical pain were evaluated six weeks post vehicle/drugs administrations orally, followed by brain biochemical measurements. TA treatment alleviated Gly-induced hyperalgesia in similar version to the values of control and AA groups by increasing significantly (p < 0.05) nociceptive thresholds. Moreover, TA-treatment significantly decreased malondialdehyde (MDA) and pro-inflammatory cytokines (TNF-α, IL-1β, and IL-6) levels, significantly increased anti-inflammatory cytokines (IL-10, IL-4, and TGF-1β) levels, and antioxidant enzymes, catalase (CAT), glutathione peroxidase (GPx), and superoxide dismutase (SOD) activities compared to Gly-treated mice (p < 0.05). Conclusively, TA treatment exerted antinociceptive and anti-inflammatory actions, possibly through its antioxidant and anti-inflammatory actions in Gly-exposed mice. Notably, TA pre-treatment showed a better response than TA and Gly co-administration. We propose the potential neuroprotective and ameliorative functions of TA in Gly-induced hyperalgesia. This merits further clinical research into protective roles of TA against pesticide-related conditions.

## Introduction

Ever since the introduction of glyphosate-based herbicide (Gly) to the market in 1974 [1], it has become the world’s most widely used herbicide [2]. Glyphosate is the major and the most active component of Gly. It is known for its potent weed-killing actions, possibly through the inhibition of the shikimate enzymatic pathway, required for aromatic amino acid (L-tryptophan, L-phenylalanine, and L-tyrosine) biosynthesis in plants [3]. It was initially conceived that glyphosate is nontoxic to higher mammals, probably due to lack of shikimate enzymatic pathway in mammals, hence production of no physiological role [4]. Even though there is no related physiological role of shikimate pathway in mammals, there have been controversies on the potential health risks of chronic exposure to glyphosate from occupation, water, food, and environmental contamination [5, 6]. However, glyphosate exposure has been implicated in the pathogenesis of several neurological disorders through stimulation of oxidative and inflammatory pathways [7]. Gly formulations usually contain the isopropylamine salt of glyphosate in mixture of unrevealed ancillary substances and surfactants which promote the stability and absorption of the compound into the plant tissues [8]. Although manufacturers of Gly have often proclaimed that the ancillary substances are inert, many reports suggest that Gly formulations may be significantly more toxic than pure glyphosate itself [9]. Worthy of note that everyone is ubiquitously exposed to glyphosate and its metabolite aminomethylphosphonic acid (AMPA) through the Gly formulations sprayed on crops or in the water body.

Regarding the potential neurotoxic effects of glyphosate, reports have shown that glyphosate freely crosses the blood brain barrier, suggesting an adverse effects of glyphosate on nervous system correlating with long-term pathogenesis of neuropsychiatric conditions [10]. Chronic and subchronic oral glyphosate exposure has been reported to induce depression-like behaviour in adult rats [11, 12], whereas, neurodevelopmental impairment associated with glyphosate exposure was reported to increase immobility time and reduced locomotor activities in young offspring [13]. Evidence is sparse thus far to evaluate the effect of chronic glyphosate exposure on pain perception. Therefore we aimed at evaluating the effect of glyphosate on pain perception in mice. Notwithstanding regulatory strategies to reduce human exposure to glyphosate, occupational or accidental exposure remains a chief concern, emphasizing the need for effective protective and therapeutic interventions to attenuate glyphosate toxicity

One of the possible avenues for intervention against glyphosate-induced neurotoxicity is through the application of potent antioxidants, such as tannic acid (TA). TA is a hydrolysable polyphenolic compound approved as food additive by US Food and Drug Administration (FDA) [14]. In the recent year, the application of TA has garnered significant attention due to its far-reaching physiological actions such as antitumor, antimicrobial, neutralization of reactive oxygen species (ROS), mitigation of oxidative stress-associated injury and anti-inflammatory actions [15-17].

In the context of glyphosate-induced neurotoxicity, oxidative stress, neuroinflammation, and alterations in neurotransmitters play a chief role in mediating its harmful effects. Exposure to glyphosate influences generation of ROS, leading to lipid peroxidation, protein oxidation, and DNA damage, essentially climaxing tissue injury and neuroinflammation [18-20]. Furthermore, glyphosate-induced oxidative stress may be implicated in nociceptive pathways, thereby contributing to increased pain perception and sensitization. On the basis of antioxidant and anti-inflammatory properties of TA, there is currently a growing interest in reconnoitering its possible neuroprotective and ameliorative efficacy against glyphosate-induced neurotoxicity. Studies in the past have demonstrated the promising results using other models of pathologies, in which TA administration mitigated oxidative stress, inflammation, and related pathological alterations [21]. Though there are accumulating evidence strengthening the antioxidant and neuroprotective functions of TA, its explicit role in glyphosate-induced hyperalgesia and neuroinflammation remain unstudied. Therefore, this preclinical evaluation aims to underpin the potential antinociceptive and anti-inflammatory, and antioxidant roles of TA in Gly-exposed mice. Elucidating some of the possible mechanisms underlying TA neuroprotective actions could offer good insights into the development of novel approaches for attenuating pesticide toxicity and related health hazards.

## Materials and Methods

### Animals

Male Swiss albino mice (weighing 25 - 30 g) used in this research were obtained from the animal husbandry of the Central Research Laboratory (CRL), University of Ilorin, Nigeria. Subsequent to procurement of the animals, they were acclimatized for 7 days and reared in wooden cages with sawdust-covered floors (8 mice/cage; 45 cm × 20 cm × 15 cm) under a sustained optimum level of hygiene, standard temperature condition (24 ± 2 °C), and light/dark cycle 12 h/12 h. All animals were supplied with food and water *ad libitum*. The experimental procedures were in accordance to the University of Ilorin Ethical Review Committee’s (UERC) guiding principles which conform to the provisions of National Institutes of Health Guide for the Care and Use of Laboratory. Efforts were made concertedly in ensuring that animals did not surfer or that suffering was minimized. This study was approved by University of Ilorin Ethical Review Committee’s (UERC) with the approval number UERC/ASN/2022/2364.

### Glyphosate Based Herbicide and other chemicals

Roundup Turbo herbicide (Monsanto Co., St. Louis, MO; Monsanto of Brazil Ltda, São Paulo, Brazil) containing glyphosate as the active ingredient was used in this study. This formulation is constituted by 450 g/L of glyphosate, 648 g/L of isopropylamine salt and 594 g/L of inert ingredients. Tannic acid and ascorbic acid used in this study were purchased from Sigma Chemical Co. (St. Luis, MO, USA) and prepared in distilled water.

### Experimental design

Forty eight (48) healthy male Swiss albino mice were randomly divided into six experimental groups equally, n=8/group (Table 1). Each group was subjected to either oral gavages by distilled water, glyphosate-based herbicide (Gly), tannic acid (TA) or ascorbic acid (AA); control (distilled water 0.2 ml/kg, *p.o.*, n = 8), Gly (administered with Gly 500 mg/kg, *p.o.*, n = 8), Pre-TA + Gly (pre-treated with TA 50 mg/kg *p.o.* for 7 days and afterwards treated with Gly 500 mg/kg, *p.o.*, n = 8), TA + Gly (treated with TA 50 mg/kg, *p.o.* co-administered with Gly 500 mg/kg, *p.o.*, n = 8), Pre-AA + Gly (pre-treated with AA 10 mg/kg *p.o.* and afterwards treated with Gly 500 mg/kg, *p.o.*, n = 8), and AA + Gly (treated with AA 10 mg/kg, *p.o.* coadministered with Gly 500 mg/kg, *p.o.*, n = 8). The glyphosate-exposed group received a Gly solution prepared with distilled water at a dose of 500 mg/kg. The chosen Gly dose used in this study references the lowest no observable adverse effect level (NOAEL) for maternal and developmental toxicity in rabbits, which according to the European Union [22], served as the reference dose for the definition of tolerable daily consumption for Gly. In the same vein, the dose of Gly was selected based on glyphosate subchronic and chronic exposures of NOAEL of 500 mg/kg/day for mice previously reported by EPA (1993) [23]. Of note is that the dose of Gly (500 mg/kg) used in this study was higher than the concentrations that human population is normally exposed to [24]. Nevertheless, the dosage agrees with what was reported by Ford et al. (2017) [25] who asserted that many toxicological studies with pesticides are often carried out at high concentrations in order to reveal the possible mechanism of action of the chemical. TA dose used in this study was selected as earlier reported [26, 27] while AA dose was also selected as previously used [28].

**Figure 1.**
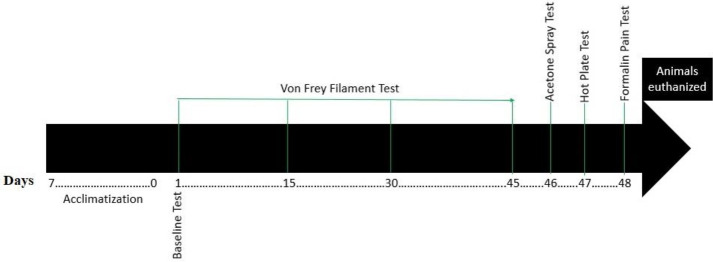
Experimental design flow chart.

All animals were sub-chronically exposed to Gly for 45 days. von Frey filament test was carried out on day 1 (for baseline assessment test), 15, 30, and 45, while acetone spray test, hot plate test, and formalin pain tests were carried out on days 46, 47, and 48 respectively (Figure 1). These behavioral tests were performed in order to evaluate responses to different types of pain stimuli. At the completion of behavioral tests on day 48, animals were euthanized to determine specific activities of anti-oxidant enzymes, neurotransmitter alteration, and pro-inflammatory/anti-inflammatory cytokines concentrations in the brain.

### Behavioral tests

#### von Frey Filament Test (VFFT)

von Frey filament test (VFT) was used to evaluate the response of the mice to mechanical pain stimulation. Briefly, animals were allowed to acclimatize to the testing room for 10 mins. Subsequently, they were placed in a confined cage having perforated mesh floor and allowed to steady and settle in a relaxed position. Paw withdrawal response was witnessed in each mouse after an unconscious application of von Frey filament touch to the planter surface of the right hind limb. The filaments were applied with the application of slight pressure to regulate it to bend when applied to paw. Animals were observed for provocation of reaction by paw withdrawal when various force rankings were applied to the paws of the animals in increasing order. The filament that provokes a reaction was observed and replaced with succeeding lower-rated filament. On the other hand, when there is no observable response, the succeeding filament was applied to ensure specific identification of minimal required force that provoked a response. When there was an alteration in the original position of the animal, the experiment was repeated for 4 times to ensure exactitude [29].

#### Acetone Spray Test

In order to carry out an investigation on cold allodynia, acetone spray test was used. The housing of animals in the von Frey filament test was also used for the acetone spray test. Animals were again acclimatized and well relaxed to the testing room before the experiment. They were subsequently habituated for 15 minutes to testing cage with a wire mesh metal floor. About 50 μL of acetone was sprayed to the planter surface of the fight hind limb after habituation. Mice were noticed for different responses; paw lifting, scratching and touching the right hind limb for 5 min. Total duration of reaction was evaluated and analyzed as cumulative reaction time [30].

#### Hot Plate Test (HPT)

Reaction to heat pain was evaluated by measuring the thermal nociceptive threshold by placing mice on a rodent hot plate having its temperature constantly sustained at 52 ± 0.2 °C. The latency of jumping, flinching, and paw licking behavior was observed and recorded. A total cut-off time of 60 s was set in order of prevent tissue damage [31, 32].

#### Formalin Pain Test

Assessment of chemical pain was investigated using formalin pain test as previously described [33]. Briefly, 0.2 ml of 5 % formalin solution was injected into the dorsal surface of the mice paw and they were afterwards returned into a testing chamber. The reactions of the animals were video recorded immediately after the animals were placed back into the testing chamber. Two phases of behavior were observed: Phase 1 which specifies the acute phase and indicated by paw licking behavior for duration 0–5 min and phase 2 which signifies tonic phase in which paw licking behavior was observed for 20 min (20-40 min) after formalin injections.

### Biochemical assays

#### Tissue preparation

Upon the completion of the neurobehavioral testing, animals were anaesthetized using intraperitoneal (i.p.) injection of Ketamine (90 mg/kg) and xylazine (7 mg/kg) before they were euthanized. Ketamine and xylazine doses used in this study were selected based on the experience in the laboratory, and fall within the recommended doses for mice as previously reported [34, 35]. After euthanasia mice medial prefrontal cortices selected for biochemical evaluation were immediately dissected and isolated. The dissected brain tissues (0.5 g) were rapidly homogenized in 3 ml of 0.1M of ice-cold phosphate buffer pH 7.4 using a Percellys Minilys Homogenizer (CAYMAN CHEMICAL, USA) by 60 s. The homogenates were subsequently centrifuged at 5000 rmp for 15 min and the supernatants were separated and kept at −80 °C until further analysis.

#### Determination of antioxidant enzymes activities

##### Measurement of lipid peroxidation in the cortex

The thiobarbituric acid reactive substance (TBARS) concentrations as an indication of malondialdehyde (MDA) production was evaluated as previously described by Ohkawa et al. (1979) [36]. MDA is an end product of lipid peroxidation and forms complex with TBA-TCA to form a coloured complex reaction as high temperature revealing as absorption maximum at 535 nm. 100 μL of normal saline was mixed with 100 μL of supernatant and 400 μL of TBA–TCA mixture, followed by incubation for 10 min in boiling water bath and then cooled at room temperature. Subsequent to centrifugation at 500 rmp for 10 min, 100 μL of supernatant was reacted with 100 μL of 0.7% TBA in cuvette and read at 535 nm. The concentration of MDA was calculated using standard curve from 1, 1, 3, 3-tetraethoxypropane and expressed in nmoles/mg.

##### Measurement of Catalase activity

The activity of catalase was evaluated by mixing 0.05 ml tissue supernatant with 3 ml hydrogen peroxide (H_2_O_2_). The absorbance activity was measured against a blank that contained 3 ml of PBS at a wavelength of 240 nm. The amount of H_2_O_2_ was proportional to the absorbance, which was reduced when catalase destroys H_2_O_2_. This is an evaluation of H_2_O_2_ breakdown and is presented as mol H_2_O_2_ broken-down per milligram of protein per minute [37].

##### Measurement of Superoxide dismutase activity (SOD)

The activity of SOD was measured using the nitroblue tetrazolium (NBT) as previously reported by Sun et al. (1988) [38]. Briefly, 50 μL of the crude enzyme extract was added to a solution containing 13 mM L -methionine, 75 μM pnitro blue tetrazolium chloride (NBT), 100 μM EDTA and 2 μM riboflavin in a 50mM potassium phosphate buffer (pH 7.8). The reaction occurred in assay tubes under 30W fluorescent lamp illumination 25 °C for 15 minutes. Using a spectrophotometer, the blue formazane produced by NBT photo-reduction was measured by the absorbance at 620 nm. There was no enzyme extract in the control reaction mixture. The blank solution was kept in the dark and had the same complete reaction solution. One SOD unit of activity was defined as the amount of enzyme needed to inhibit 50% of NBT photo-reduction relative to tubes lacking the tissue extract. Activity was calculated as units (U) per mg soluble protein per min (U mg−1 protein min−1).

##### Measurement of Glutathione peroxidase (GPx) activity

The oxidation of glutathione by the GPx cumene hydroperoxide is catalyzed by the GPx. In the presence of glutathione reductase and NADPH, the oxidized glutathione is converted back to regenerative glutathione. In this study, the NADP+ obtained at 340 nm was evaluated [39].

#### Enzyme-linked immunosorbent assay (ELISA)

The NF-κB, PGE_2_, Pro-inflammatory cytokines (TNF-α, IL-1β, and IL-6), anti-inflammatory cytokines (IL-4, IL-10, and TGF-ß1) and neurotransmitters (dopamine and noradrenaline) ELISAs were carried out following the manufacturer’s instructions. Subsequent to homogenizing an appropriate quantity of brain tissues (0.5 g) and recovery of supernatant after centrifugation (at 5000 rmp/15 min), the total concentration of protein in each group was measured with the application of BCA method. Protein samples were complexed with antibodies supplied in the corresponding kits using a 96-well plate. The absorbance values were evaluated using a microplate reader. Concentrations were then adjusted to total protein content. All procedures were repeated three times [40].

### Histological analysis

Sample of the medial prefrontal cortex were fixed using 10% phosphate buffered formalin for 72 h. Following established protocol of Bancroft and Gamble (2008) [41], microscopic examination of the tissues were carried out using 4–5 μm sections stained with hematoxylin and eosin. The slides were appropriate coded before a light microscope (Leica DM 500, Germany) examination and image capturing with the aid of digital camera (Leica ICC50 E, Germany).

### Statistical analyses

Data obtained from this study were analyzed using one-way and two-way analysis of variance and Tukey’s post hoc test with GraphPad PRISM 9 software (GraphPad PRISM 9.1.1 Software (San Diego CA, USA)). Values of p < 0.05 were considered significant.

## Results

### Tannic acid reduces pain perception in mechanical, thermal and chemical pain stimulations

#### von Frey Experiment

**Figure 2.**
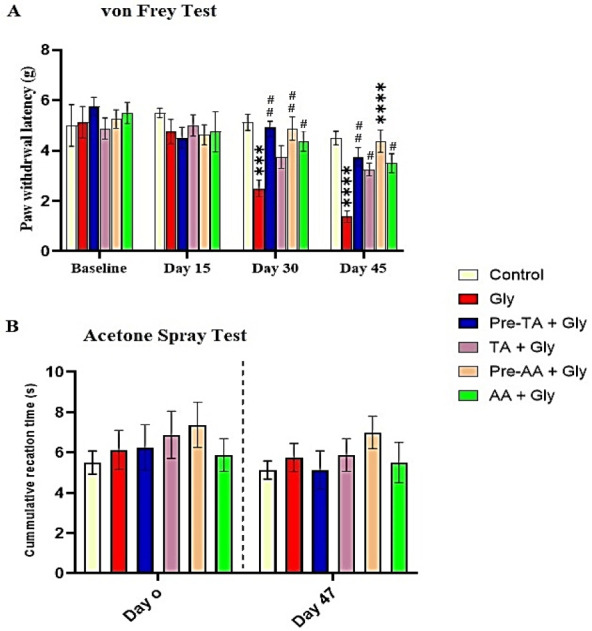
Effects of tannic acid (TA) on pain perception using mechanical pain stimulation and acetone spray test following oral Gly administration. (A) von Frey Filament Test (B) Acetone Spray Test. Data are reported as mean ± S.E.M (n = 8 animals per group). *P < 0.05, **P<0.01, ***P < 0.001, ****P < 0.0001 verses control. #P < 0.05, ##P < 0.01, ###P < 0.001, ####P < 0.0001 verses Gly. Two-way ANOVA followed by Tukey post hoc test.

The effect of TA was tested on mechanical pain perception using von Frey testing paradigm. As shown in Figure 2A, in the glyphosate-based herbicide treated group (Gly), mechanical pain stimulation using von Frey filament on days 30 and 45 of the experiment resulted into a significant decrease in the pain threshold as demonstrated by a significant decrease (P < 0.001) in the paw withdrawal latency relative to the control group. However, TA pre-treatment (Pre-TA +Gly group) decreased pain perception as displayed by a significant increase in the paw withdrawal latency relative to the Gly group, an effect similar to that obtained in the groups treated with ascorbic acid (AA). Interestingly, co administration of TA and Gly manifested no significant difference in the paw withdrawal latency on day 30 of the experiment relative to Gly. This group however, showed a significant increase (p > 0.05) in the paw withdrawal latency relative to Gly group on day 45.

#### Acetone Spray Test

Cold hyperalgesia was evaluated by application of 50 μL acetone to the planter surface of the hind paw of the animals. Results of the acetone experiment (Figure 2B) showed that cold pain sensitivity in Gly group was not statistically different compared to the control group and the treated mice both at baseline level and on the 47th day of the experiment.

#### Hot Plate Test

**Figure 3.**
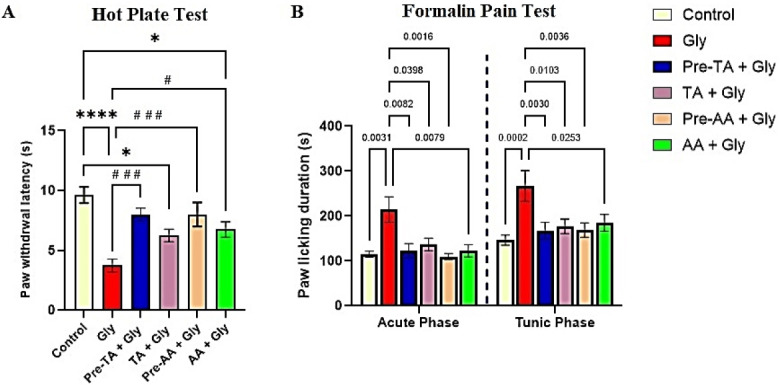
Effects of tannic acid (TA) on pain perception using thermal pain stimulation and chemical pain stimulation following oral Gly administration. (A) Hot Plate Test (B) Formalin Pain Test. Data are reported as mean ± S.E.M (n = 8 animals per group). *P < 0.05, **P < 0.01, ***P < 0.001, ****P < 0.0001 verses control. #P < 0.05, ##P < 0.01, ###P < 0.001, ####P < 0.0001 verses Gly. Two-way ANOVA followed by Tukey post hoc test

According to Figure 3A, the responsiveness of Gly group to thermal heat stimulation was significantly reduced (P < 0.0001) compared to the control group, indicating heat hyperalgesia in the Gly group. Interestingly, pre-treatment with TA before Gly exposure as shown in the Pre-TA + Gly group showed a significantly increased (P < 0.001) paw withdrawal latency (decreased pain perception), an effect similar to that obtained in the control group and pre-treatment with AA group. Furthermore, co-administration of TA with Gly as shown in the TA + Gly group showed no significant alteration in the responsiveness of the mice to heat pain stimulation compared to the Gly. An effect that was different when Gly group is compared with AA + Gly group. In this instance, co administration of the standard drug AA with Gly offered a significant increase in the paw withdrawal threshold relative to the Gly group. Put to together, the anti-nociceptive activity of TA is apparent in pre-treatment of TA in Gly exposed mice compared to the co-administration of TA with Gly.

#### Formalin Pain Test

In order to demonstrate nocifensive responsiveness to chemical pain stimulation, intraplanter injection of 0.2 ml of 5 % formalin solution was performed on the mice hind paw (Figure 3B). Injection of the formalin solution caused acute nociceptive formalin reaction which lasted for 5 min (acute phase), then led to chronic inflammatory response, which began about 20 min to 40 min post formalin administration (tunic phase). In the acute phase of the experiment, exposure to glyphosate as demonstrated in the Gly group showed a significant increase (P < 0.01) in the licking time during the acute phase of formalin injection compared to the control group. Conversely, both Pre-TA + Gly and TA + Gly groups manifested a significantly decreased (P < 0.01 and P < 0.05 respectively) paw licking duration compared to untreated Gly exposed group, this effect was similar to effects of the standard drug both at pre-treatment and co-administration groups. In the tunic phase of the experiment, the paw licking duration in the Gly was significantly increased (p < 0.001) in the Gly group relative to the control group, an indication of inflammatory process. The effects of TA both in Pre-TA + Gly and TA + Gly groups were similar to what was observed in the acute phase of the experiment.

### Effects of TA on Gly-induced alterations in antioxidant enzymatic activity and lipid peroxidation

**Figure 4.**
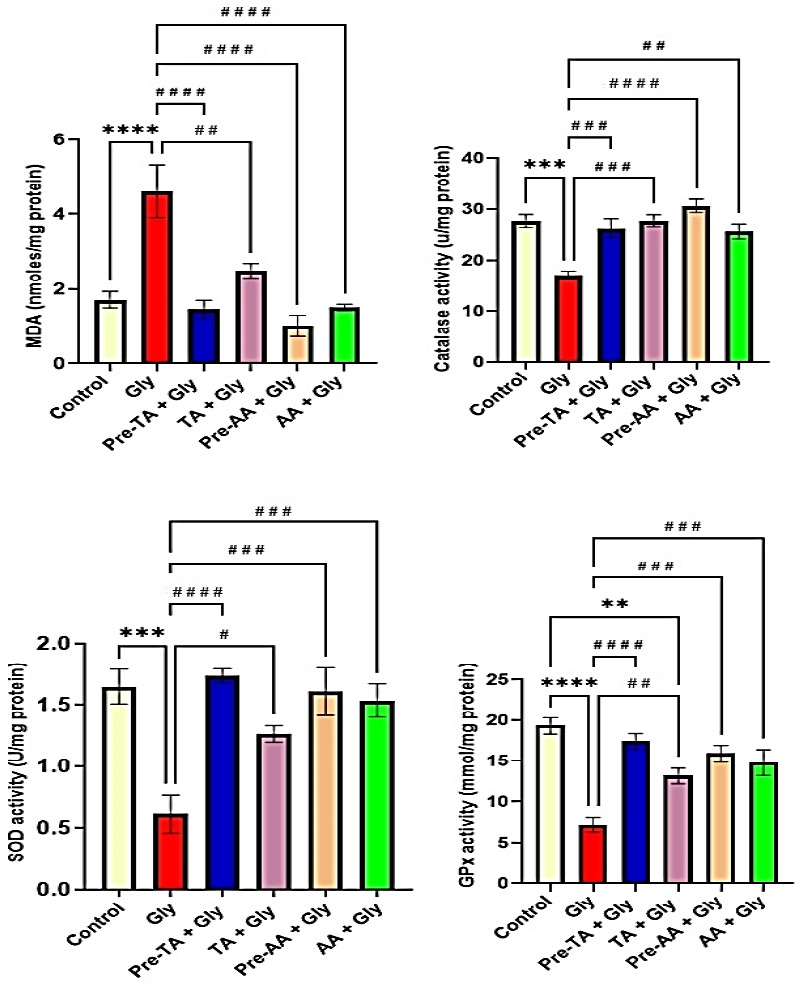
Effects of tannic acid (TA) on antioxidant enzymes activities following oral Gly administration. (A) Medial prefrontal cortex malondialdehyde (MDA) levels (B) Medial prefrontal cortex catalase activity (C) Medial prefrontal cortex superoxide dismutase activity (SOD) (D) Medial prefrontal cortex glutathione peroxidase (GPx) activity. Data are reported as mean ± S.E.M (n = 8 animals per group). *P < 0.05, **P < 0.01, ***P < 0.001, ****P < 0.0001 verses control. #P < 0.05, ##P < 0.01, ###P < 0.001, ####P < 0.0001 verses Gly. One-way ANOVA followed by Tukey post hoc test.

The activities of antioxidant enzymes, catalase, SOD, and GPx as well as MDA concentrations have been shown in Figure 4. Gly exposure increased lipid peroxidation as the prefrontal cortex level of MDA was significantly increase (P < 0.0001) in the Gly group compared to the control. However, TA pre-treatment (Pre-TA + Gly group) and coadministration of TA with Gly (TA + Gly group) ameliorated these effects by significantly reducing (P < 0.0001 and P < 0.01 respectively) the concentration of MDA in the prefrontal cortex of Gly-exposed mice. The observed effects of TA-treated groups were similar to the control and the AA-treated groups (Figure 4A). Furthermore, Gly group manifested a significantly reduced activities of catalase enzyme (P<0.001, Figure 4 B), SOD enzyme (P < 0.001, Figure 4C), and GPx enzyme (P<0.0001. Figure 4 D) compared to the control group. Notably, TA pre-treatment (Pre-TA + Gly group) and co-administration of TA with Gly (TA + Gly group) respectively mitigated these effects by significantly increasing the activities of catalase enzyme (P < 0.001 and (P < 0.001), SOD enzyme (P < 0.0001 and P < 0.05), and GPx enzyme (P < 0.0001 and P < 0.01). These effects observed to be similar to the control group and groups treated with AA (Figure 4B-C).

### TA abated Gly-induced neuro-inflammation

**Figure 5.**
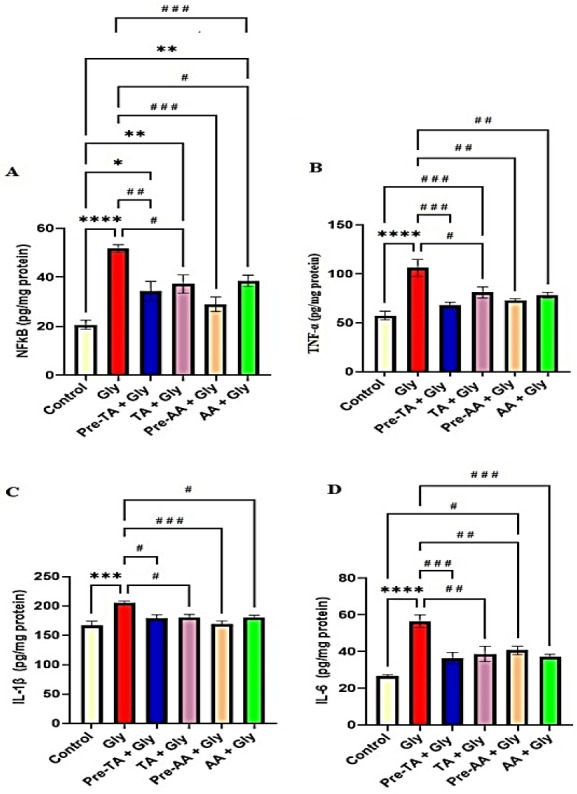
Effects of tannic acid (TA) on medial prefrontal cortex pro-inflammatory cytokine concentration following oral Gly administration. (A) Nuclear factor kappa B (NF-κB) (B) Tumor Necrosis Factor Alpha (TNF-α) (C) Interleukin-1β (IL-1β) (D) Interleukin 6 (IL-6). Data are reported as mean ± S.E.M (n = 5 animals per group). *P < 0.05, **P < 0.01, ***P < 0.001, ****P < 0.0001 verses control. #P < 0.05, ##P < 0.01, ###P < 0.001, ####P < 0.0001 verses Gly. One-way ANOVA followed by Tukey post hoc test.

Results on the influence of TA pre-treatment or co-treatment with Gly on the biomarkers of inflammation are displayed in Figure 5A–D. In comparison with the control group, exposure to Gly per se significantly increased NF-κB (p < 0.0001), TNF-α (p < 0.0001), IL-1β (P < 0.001), and IL-6 (p < 0.0001) concentrations in the prefrontal cortex of the exposed mice. TA pre-treatment (Pre-TA + Gly group) and co-administration of TA with Gly (TA + Gly group) did not significantly alter these indices of inflammation in the treated mice when compared to the control group and AA treated groups. Conversely, the increased concentrations of NF-κB, TNF-α, IL-1, and IL-6 observed in the Gly group were markedly abrogated when compared to TA pre-treatment (Pre-TA + Gly group) and co-administration of TA with Gly (TA + Gly group).

### Effects of TA on anti-inflammatory cytokines levels in Gly-exposed mice

**Figure 6.**
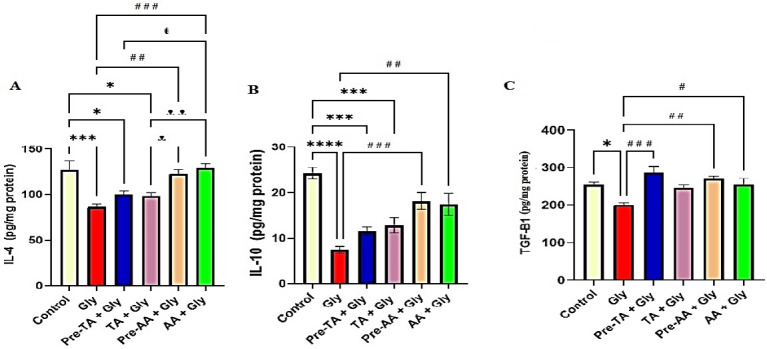
Effects of tannic acid (TA) on medial prefrontal cortex anti-inflammatory cytokine concentration following oral Gly administration. (A) Interleukin 4 (IL-4) (B) interleukin 4 (IL-10) (C) Transforming growth factor-β (TGF-β1) Test. Data are reported as mean ± S.E.M (n = 5 animals per group). *P < 0.05, **P < 0.01, ***P < 0.001, ****P < 0.0001 verses control. #P < 0.05, ##P < 0.01, ###P < 0.001, ####P < 0.0001 verses Gly. ŧ P < 0.05, ŧ ŧ P < 0.01, ŧ ŧ ŧ P < 0.001, ŧ ŧ ŧ ŧ P < 0.0001 verses Pre-TA + Gly. ᴥ P < 0.05, ᴥ ᴥ P < 0.01, ᴥ ᴥ ᴥ P < 0.001, ᴥ ᴥ ᴥ ᴥ P < 0.0001 versus TA + Gly. One-way ANOVA followed by Tukey post hoc test.

Data on the effects of TA on anti-inflammatory cytokines are shown in Figure 6A–C. Results show that exposure to Gly caused a significant decrease in the concentrations of anti-inflammatory cytokines IL-4 (p < 0.001), IL-10 (p < 0.0001), and TGF-1β (p < 0.05) compared to the control group. TA pre-treatment (Pre-TA + Gly group) and co-administration of TA with Gly (TA + Gly group) did not significantly alter the concentrations of IL-4 and IL-10 cytokine in the treated mice when compared to the Gly group. Conversely, TA pre-treatment (Pre-TA + Gly group) significantly reduced the concentration of TGF-1β (p < 0.0001) while co-administration of TA with Gly (TA + Gly group showed no significantly alteration compared to the Gly group. Of note, treatment with standard drug, AA marked increased the concentrations of the anti-inflammatory cytokines in the prefrontal cortex of Gly exposed mice.

### TA mitigated alterations of pain mediators in Gly-exposed mice

**Figure 7.**
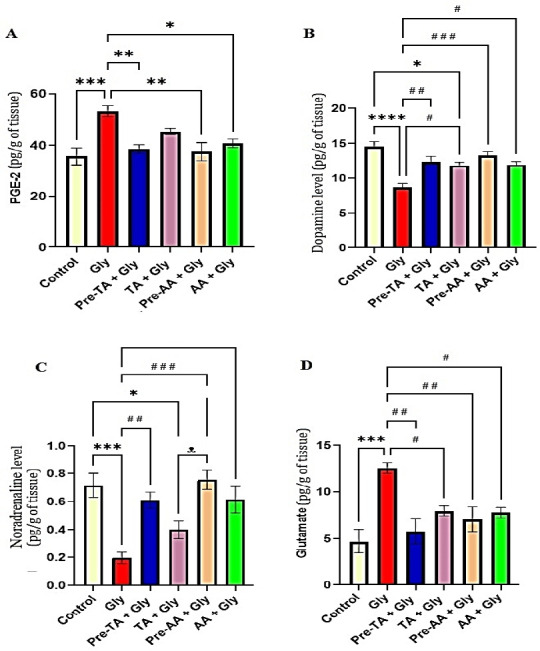
Effects of tannic acid (TA) on medial prefrontal cortex pain mediators following oral Gly administration. (A Prostaglandin E2 (PGE2) (B) Dopamine concentration(C) Noradrenalin concentration. Data are reported as mean ± S.E.M (n = 58 animals per group). *P < 0.05, **P < 0.01, ***P < 0.001, ****P < 0.0001 verses control. #P < 0.05, ##P < 0.01, ###P < 0.001, ####P < 0.0001 verses Gly. ᴥ P < 0.05, ᴥ ᴥ P < 0.01, ᴥ ᴥ ᴥ P < 0.001, ᴥ ᴥ ᴥ ᴥ P < 0.0001 versus TA + Gly. One-way ANOVA followed by Tukey post hoc test.

PGE_2_, dopamine, noradrenalin, and glutamate contribute largely to the mediation of pain through central enhancement of central sensitization. Figure 7A–D shows the effects on TA on pain mediators in Gly-exposed mice. Brian level of PGE_2_ was markedly increased (P < 0.001) in Gly-exposed mice compared to the control group. There was no significant alteration in the brain level of PGE_2_ of the TA pre-treatment, Pre-TA + Gly group in comparison with the control and the standard drug AA-treated mice. Essentially, TA pre-treatment (Pre-TA + Gly group) significantly reduced (p < 0.01) the brain PGE_2_ concentration while co-administration of TA with Gly (TA + Gly group) showed no remarkable alteration compared to the Gly group. There was a significant reduction in the brain concentrations of dopamine (p < 0.0001) and noradrenalin (p < 0.001), and significant increase (p < 0.001) in the brain glutamate levels of Gly group in comparison with the control. Treatments with TA manifested similar effects relative to the control and treatment with the standard drug, AA. Nevertheless, TA pre-treatment (Pre-TA + Gly group) displayed marked increase in the levels of dopamine (p < 0.01) and noradrenalin alongside a significant decrease in the concentration of glutamate (p < 0.01 compared with Gly group. The effect of co-administration of TA with Gly (TA + Gly group) was relatively variant. This group showed no significant difference in the level of noradrenalin, but significantly increased dopamine level (p < 0.05) and significantly decreased glutamate (p < 0.05) concentration compared to the Gly group.

### Effect of TA treatment on medial prefrontal cortex histological features of Gly-exposed mice.

**Figure 8.**
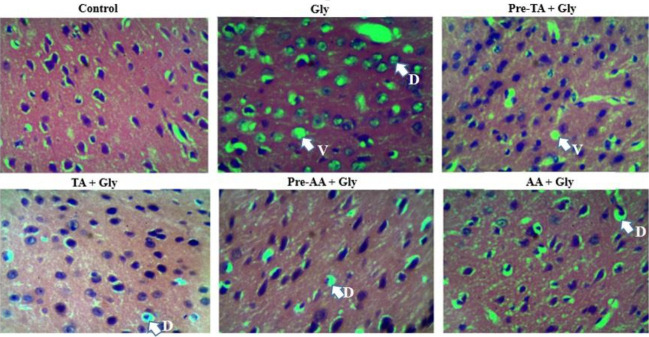
Representative histopathological appearance of medial prefrontal cortex between experimental groups. Normal tissue architecture (Control). Numerous degenerated neurons (nuclei dissolution and vacuolation) characterized by altered shape, staining shrinkage, and chromatolysis (Gly). Similar tissue histology compared to the control, although marked with negligible neuronal vacuolation (Pre-TA + Gly). There were reduced degenerative neurons as evidenced by significant reduction in nuclei dissolution and vacuolation (TA + Gly). Similar histological appearance compared to the control, although, there was a presence in nuclei dissolution (Pre-AA + Gly). Decreased degenerative neurons with negligible nuclei dissolution and vacuolation (AA + Gly). D = Dissolution of nucleus, V = Vacuolation. (H & E stain, × 400) (n = 3).

The effect of TA on the histology of medial prefrontal cortex after mice were exposed to Gly for 45 days is shown in Figure 8. Exposure of the mice to Gly resulted into significant distortion in the histoarchitecture of the medial prefrontal cortex. However, treatment of mice with TA (TA pre-treatment, Pre-TA + Gly group or co-administration of TA with Gly, TA + Gly group) caused a significant reversal of the distorted histoarchitecture of the medial prefrontal cortex.

## Discussion

Excessive exposure to pesticides from occupation, water, food substances, and air has been associated with pathogenesis of numerous neurological conditions both in humans and animals [42]. The major causative features to the deleterious effects of pesticides involve their persistence, presence of their residues in food components, and bioaccumulation properties [43]. Among the most globally used pesticides, glyphosate-based herbicides (Gly) top the list [44]. Indeed, the results from this study revealed that even at no observable adverse effect level (NOAEL) dose of Gly, subchronic oral exposure to Gly was capable of inducing hyperalgesia as demonstrated through mice in this study, as well as provoking oxidative stress and neuro-inflammation in the medial prefrontal cortex of the mice. The adoption of NOAEL (500 mg/kg) of Gly used in this study was based on subchronic feeding exposure study where a relatively low dose of commercial formulation of Gly was sued to consider the systemic toxicity of Gly consumption and its effects on animals weight gain [23].

While previous reports on Gly revealed that continuous exposure to Gly even at low dose resulted into development of motor dysfunction and mood disorders, such as anxious and depressive-like behavior [45, 46], the findings in the current study revealed for the first time, that Gly exposure increases pain perception in mice. Throughout this study except in cold pain stimulation, it was observed that exposure to Gly provoked a marked alteration in responsiveness of mice to pain stimuli as manifested in decreased paw withdrawal thresholds and increased paw licking duration in the different pain neurobehavioural paradigms used in this study. These findings are suggestive of dyshomeostasis in pain transmission and perception on account of Gly exposure. The findings also corroborate the affirmations in a clinical study that there is a high risk of numbness or prickling symptoms being prevalent among farmer who are chronically exposed to pesticides in China [47].

Cellular antioxidant defense mechanisms play a crucial role in the suppression of oxidative stress and maintenance of constant internal environment by preservation of cellular redox reaction [48]. The hyperalgesia observed in the Gly exposed group in this study could be related to altered oxidative stress parameters in the mice medial prefrontal cortex. Hacimuftuoglu et al (2006) [49] had previously reported that oxidative stress induces hyperalgesia, hence the decrease in the activities of antioxidant enzymes in this study may partly explain the decreased in the pain threshold of animals exposed to Gly. The capacity of Gly to influence the generation of an oxidizing environment in the brain through reactive oxygen species has been reported [50], as Gly is lipophilic and has the capacity to cross the blood brain barrier to produce numerous deleterious effects in the brain [51].The significant reduction in the activities of prefrontal cortex antioxidant enzymes including catalase, SOD, GPx alongside increased concentration of MDA in Gly exposed mice indicates impairment in the antioxidant defense mechanisms which in turn may cause accretion of cytotoxic free radical with impaired physiological processes, possibly including the neuroinflammation and hyperalgesia observed in this study. The medial prefrontal cortex is highly involved in pain processing. Dyshomeostasis in this region of the brain on account of oxidative stress and neuroinflammation has been implicated in hyperalgesia in humans and animals [52]. This is relevant because it reveals the susceptibility of the medial prefrontal cortex to maintain antioxidant enzyme activities in the presence of Gly exposure.

The contributions of neuro-inflammation to Gly-induced neurotoxicity was also evaluated by assessing inflammatory markers, such as NF-κB, TNF-α, IL-1β, and IL-6. Elevation in the concentration of NF-κB has been associated with increased oxidative stress through high production of cyclooxygenase-2 (COX-2) and inducible nitric oxide synthase (iNOS) which stimulates the production reactive oxygen species in cells (Kumar et al., 2021). NF-κB also enhances neuroinflammation by activating glial cells (microglia and astrocytes) and neurons to increase production of proinflammatory cytokines such as TNF-α, IL-1β, IL-6 and other inflammatory mediators [53]. Whereas TNF-α is considered as the chief regulator of inflammatory reaction due to its role in recruiting immune cells to the site of injury, thereby intensifying the neuroinflammatory reaction. Increased production of pro-inflammatory cytokines play a key role in the development and maintenance of hyperalgesia through peripheral receptors sensitization, promotion of central sensitization, and enhancement of neuronal excitability [54]. The marked elevation of inflammatory markers in the medial prefrontal cortex of Gly-intoxicated mice connotes their contributions to increased pain perception observed in the Gly-exposed mice.

Furthermore, anti-inflammatory cytokines such as interleukin-4 (IL-4), interleukin-10 (IL-10) and transforming growth factor beta-1 (TGF- β1), act by suppressing the production and activities of pro-inflammatory cytokines alongside their signaling pathways [55]. Moreover, studies have shown that anti-inflammatory cytokines have capacity to directly inhibit the peripheral nociceptors sensitization and pain signals transmission [56]. For instance, IL-10 has been reported to mitigate sensitization of pro-inflammatory cytokines on the excitability of nociceptors thereby halting pain sensitization during inflammatory conditions [57]. In line with this, the decreased levels of the anti-inflammatory cytokines observed in the Gly-intoxicated mice according to our findings further points to an unresolved neuroinflammation which in effect resulted into an increased pain perception manifested by the animals.

Prostaglandin E2 (PGE_2_), one of the products of neuroinflammation plays an important role in pain perception, specifically in the context of inflammatory pain. PGE_2_ elicits its action by increasing the excitability of receptors expressed by neurons in the brain, influencing the central sensitization process [58]. Central sensitization involves central nervous system amplification of Pain signals, resulting into the development of un-resolving pain state and heightened pain perception. Indeed, heightened medial prefrontal cortex levels of PGE_2_ observed in this study is apparent in contributing to the actuation of pain sensitization system, leading to hyperalgesia manifested by mice exposed to Gly.

Dopamine, noradrenaline, and glutamate are neurotransmitters that participate in pain perception modulation. Although, the primary function of these neurotransmitters are associated with reward, regulation of mood, and synaptic transmission, they also facilitate pain signal processing in the nervous system [59]. Studies have shown that dysregulation of dopamine signaling often occurs in chronic pain states, resulting into altered pain processing and hyperalgesia [60]. Dopamine receptors are expressed in the prefrontal cortex where they participate in pain processing. Essentially, dyshomeostasis in dopamine signaling can facilitate modulation of pain pathways, such as the descending pain modulatory system, leading to increased pain signals and heightened pain sensitivity [61]. A decreased level of dopamine manifested by the Gly-exposed mice connotes the contribution of low concentration of dopamine to hyperalgesia reported in this study. In the same vein, noradrenalin has been shown to play key role in modulating pain perception. A reduction in the concentration of noradrenalin often results into a reduced pain inhibition due to the involvement of noradrenalin in the descending pain modulation pathway. Moreover, noradrenalin modulates neuronal activities in brain areas that participate in pain processing, such as the insula and the anterior cingulate cortex. Essentially, a decreased level of noradrenalin in the medial prefrontal cortex may heighten sensitivity to pain stimuli. Because of the role of medial prefrontal cortex in regulating emotion, a low level of noradrenalin is this region of the brain is as well attributed to heightened emotional reaction to pain and affect attentional processing of pain, where attention is focused more on pain stimuli. These assertions were further reinforced by a Kaur et al (2021) [62], who reported increased pain perception in reserpine-induced musculoskeletal pain on account of decreased brain concentration of noradrenalin. In line with this, the hyperalgesia condition experienced by the animals exposed to Gly in this study might partly be associated with decreased concentration in noradrenalin concentration in the medial prefrontal cortex of the mice.

Importantly, NMDA receptors and AMPA receptors are glutamate receptors found to be abundant in regions of the spinal cord and the brain involved in pain processing, such as the medial prefrontal cortex [63]. Release of glutamate from nociceptive neurons stimulates the glutamatergic postsynaptic receptors, leading to neuronal depolarization and increased pain transmission to the high centers [64]. Notably, hyper-secretion of glutamate or dysregulation of the signaling pathway of glutamate can as well result into neuronal hyper-excitability, maladaptive synaptic plasticity, and pain signals amplification, which in effect contribute to increased pain perception. Osikowicz et al (2013) [65] previously reported that elevated glutamatergic neurotransmission in the CNS is a common phenomenon and contributes to hyperalgesia in neuropathic pain. Increase neuroinflammation has been linked with glutamate excitotoxicity. Specifically, TNF-α has been reported to potentiate glutamate-mediated cytotoxicity [66]. Of note, Gly exposure has been attributed to neuroinflammation as well as glutamate excitotoxicity [67]. In agreement with this, we observed in our study that all the animals exposed to Gly manifested hyperalgesia behavior and essentially had an increased concentration of glutamate in their medial prefrontal cortex. This further reinforces the contribution of Gly exposure to hyperalgesia behavior noted in this study. Previous reports have shown that organophosphate exposure induces glial activation which in turn increases the concentrations of pro-inflammatory markers [68]. In the case of Gly, this effect is connected with glutamate excitotoxicity and oxidative stress resulting into hyperalgesia and neurodegeneration whose consequences are substantiated through alterations in the histological patterns of the medial prefrontal cortex.

Histological evaluations also revealed the evidence of alterations in the structure of the medial prefrontal cortex. Degenerative alterations were evidenced with medial prefrontal cortex neuronal shrinkage and perineuronal vacuolations, necrosis and chromatolysis in animals exposed to Gly when compared to the medial prefrontal cortex sections of the control group. This indeed indicates exposure to Gly-related neurotoxicity. This report agrees with the finding of previous report on Gly that exposure to Gly induces numerous behavioral and cognitive dysfunction closely related with significant histological, neurochemical and molecular impairments tissue damage [46].

Seeing the deleterious effects of Gly exposure as reported in the literature, we opted to analyze the potential antinociceptive, anti-inflammatory, and antioxidant actions of TA, a polyphenol in Gly exposed mice. TA has been previously reported to have anti-inflammatory action in formalin-induced paw edema model of inflammation in rats [27] and an antioxidant properties in paraquat-induced oxidative stress in mice [15]. In consonance with these two properties of TA, we observed that administration of TA was able to ameliorate hyperalgesia behavioral in the Gly exposed mice by protecting against neuroinflammation and oxidative stress. In the pain neurobehavioral paradigms, the TA pre-treated group showed total recovery on the test criteria while co-administration of Gly and TA group either showed no beneficial effects or partial recovery effect when compared with the control and the standard drug, AA. The neuroprotective role of TA has been reported previously using different animal models and pathologies [21]. This demonstrates the capacity of TA to as well proffer anti-nociceptive by restoring antioxidant levels, resolving neuroinflammation and mitigating altered neurotransmitters in Gly exposed mice.

Specifically, our findings on the anti-oxidant enzymes revealed that pre-treatment with TA resulted in elevation of antioxidant enzymes equivalent to those of the control group as well as reduction in the MDA levels, resulting in improvement in oxidative status. The considerable increase in the activities of catalase, SOD, and GPx alongside reduction in the concentration of MDA in the prefrontal cortex of mice pre-treated with TA or TA co-treated with Gly connotes the neuroprotective influence against oxidative damage provoked by Gly in the mice. Furthermore, we observed mice exposed to Gly exhibited heightened levels of pro-inflammatory cytokines alongside an altered concentrations dopamine, noradrenalin, and glutamate. Neuro-inflammation appears to play a pivotal role in glyphosate induced hyperalgesia, yet if not controlled, it could lead to further cell damage. Notably, the levels of the IL-4, IL-10, and TGF-β cytokine were considerably decreased in the Gly group. This is suggestive of an inhibitory action of Gly on anti-inflammatory cytokines. Hence, the increased concentrations of IL-4, IL-10, and TGF-β upon administration of TA points to a beneficial response. Moreover, treatment with TA decreased also the inflammatory biomarkers levels, restored the concentrations of the neurotransmitters involved in mediation of pain to homeostatic values. These actions indicate the therapeutic and neuroprotective effects of TA in brain tissues of mice exposed to Gly.

## Conclusions

In this study, we showed in evidence the involvement of Gly in inducing hyperalgesia which could be related to oxidative stress, neuro-inflammation, and dyshomeostasis of neurotransmitters involved in pain mediation in the prefrontal cortex of mice sub-chronically exposed to Gly. Collectively, either TA pretreatment or co-administration of TA with Gly abated Gly-induced hyperalgesia through mechanisms involving mitigation of oxidative stress, inflammation, and improvement of neurotransmitters involved in pain processing in the medial prefrontal cortex. Notable, mice pre-treated with TA were observed to be well protected against Gly-induced hyperalgesia than concomitantly treated with TA and Gly. Based on this premise, TA may be a prospective and therapeutic candidate for neurotoxicity involving pesticide, especially glyphosate-based herbicides. Clinical studies are therefore required to further validate the neuroprotective roles of TA against pesticide intoxication.

## Figures and Tables

**Table 1.** Animal groupings and treatments.

S/N	GROUP	DESCRIPTION
1	Control	Mice were orally treated with 0.2 ml/kg distilled water
2	Gly	Mice were orally treated with 500 mg/kg Gly
3	Pre-TA + Gly	Mice were orally pre-treated with 50 mg/kg tannic acid (TA) for 7 days and subsequently treated with 500 mg/kg Gly orally
4	TA + Gly	Mice were treated with co-administration of 50 mg/kg TA and 500 mg/kg Gly orally
5	Pre-AA + Gly	Mice were orally pre-treated with 10 mg/kg Ascorbic acid (AA) for 7 days and subsequently treated with 500 mg/kg Gly orally
6	AA + Gly	Mice were treated with co-administration of 10 mg/kg AA and 500 mg/kg Gly orally

Ascorbic acid (AA) was used as a standard drug.

## References

[1] Carvalho FP (2020). Glyphosate, the herbicide that become a nightmare and the Precautionary Principle. International Journal of Environmental Studies.

[2] Martins-Gomes C, Silva TL, Andreani T, Silva AM (2022). Glyphosate vs. glyphosate-based herbicides exposure: A review on their toxicity. J Xenobiot.

[3] Vicini JL, Jensen PK, Young BM, Swarthout JT (2021). Residues of glyphosate in food and dietary exposure. Compr Rev Food Sci Food Saf.

[4] Costas-Ferreira C, Durán R, Faro LR (2022). Toxic effects of glyphosate on the nervous system: a systematic review. Int J Mol Sci.

[5] von Ehrenstein OS, Ling C, Cui X, Cockburn M, Park AS, Yu F (2019). Prenatal and infant exposure to ambient pesticides and autism spectrum disorder in children: population based case-control study. BMJ.

[6] Chávez-Reyes J, Gutiérrez-Reyes CD, Hernández-Cuellar E, Marichal-Cancino BA (2024). Neurotoxicity of glyphosate: Focus on molecular mechanisms probably associated with alterations in cognition and behavior. Environmental Toxicology and Pharmacology.

[7] Lanzarin G, Venâncio C, Félix LM, Monteiro S (2021). Inflammatory, oxidative stress, and apoptosis effects in zebrafish larvae after rapid exposure to a commercial glyphosate formulation. Biomedicines.

[8] Szepanowski F, Szepanowski LP, Mausberg AK, Albrecht P, Kleinschnitz C, Kieseier BC (2018). Differential impact of pure glyphosate and glyphosate-based herbicide in a model of peripheral nervous system myelination. Acta Neuropathol.

[9] Agostini LP, Dettogni RS, Dos Reis RS, Stur E, Dos Santos EVW, Ventorim DP (2020). Effects of glyphosate exposure on human health: Insights from epidemiological and in vitro studies. Sci Total Environ.

[10] Luna S, Neila LP, Vena R, Borgatello C, Rosso SB (2021). Glyphosate exposure induces synaptic impairment in hippocampal neurons and cognitive deficits in developing rats. Arch Toxicol.

[11] Bali YA, Ba-Mhamed S, Bennis M (2017). Behavioral and immunohistochemical study of the effects of subchronic and chronic exposure to glyphosate in mice. Front Behav Neurosci.

[12] Bali YA, Kaikai NE, Ba-M’hamed S, Bennis M (2019). Learning and memory impairments associated to acetylcholinesterase inhibition and oxidative stress following glyphosate based-herbicide exposure in mice. Toxicology.

[13] Cattani D, Cesconetto PA, Tavares MK, Parisotto EB, De Oliveira PA, Rieg CEH Developmental exposure to glyphosate-based herbicide and depressive-like behavior in adult offspring: Implication of glutamate excitotoxicity and oxidative stress. Toxicology.

[14] Al-Hijazeen M, Lee EJ, Mendonca A, Ahn DU (2016). Effects of tannic acid on lipid and protein oxidation, color, and volatiles of raw and cooked chicken breast meat during storage. Antioxidants(Basel).

[15] Choi JM, Han J, Yoon BS, Chung JH, Shin DB, Lee SK (2006). Antioxidant properties of tannic acid and its inhibitory effects on paraquat-induced oxidative stress in mice. Food Science and Biotechnology.

[16] Gülçin İ, Huyut Z, Elmastaş M, Aboul-Enein HY (2010). Radical scavenging and antioxidant activity of tannic acid. Arabian Journal of Chemistry.

[17] Sahiner N, Sagbas S, Sahiner M, Silan C, Aktas N, Turk M (2016). Biocompatible and biodegradable poly(Tannic Acid) hydrogel with antimicrobial and antioxidant properties. Int J Biol Macromol.

[18] Jasper R, Locatelli GO, Pilati C, Locatelli C (2012). Evaluation of biochemical, hematological and oxidative parameters in mice exposed to the herbicide glyphosate-Roundup®. Interdiscip Toxicol.

[19] Kašuba V, Milić M, Rozgaj R, Kopjar N, Mladinić M, Žunec S (2017). Effects of low doses of glyphosate on DNA damage, cell proliferation and oxidative stress in the HepG2 cell line. Environ Sci Pollut Res Int.

[20] Wang X, Lu Q, Guo J, Ares I, Martínez M, Martínez-Larrañaga MR (2022). Oxidative stress and metabolism: A mechanistic insight for glyphosate toxicology. Annu Rev Pharmacol Toxicol.

[21] Ashafaq M, Tabassum H, Parvez S (2017). Modulation of behavioral deficits and neurodegeneration by tannic acid in experimental stroke challenged Wistar rats. Mol Neurobiol.

[22] Székács A, Darvas B (2018). Re-registration challenges of glyphosate in the European Union. Frontiers in Environmental Science.

[23] Alvarez-Moya C, Silva MR, Ramírez CV, Gallardo DG, Sánchez RL, Aguirre AC (2014). Comparison of the in vivo and in vitro genotoxicity of glyphosate isopropylamine salt in three different organisms. Genet Mol Biol.

[24] Solomon KR (2016). Glyphosate in the general population and in applicators: a critical review of studies on exposures. Crit Rev Toxicol.

[25] Ford B, Bateman LA, Gutierrez-Palominos L, Park R, Nomura DK (2017). Mapping proteome-wide targets of glyphosate in mice. Cell Chem Biol.

[26] Al-Salih RM (2010). Clinical experimental evidence: Synergistic effect of gallic acid and tannic acid as antidiabetic and antioxidant agents. Thi Qar Med J.

[27] Soyocak A, Kurt H, Cosan DT, Saydam F, Calis IU, Kolac UK (2019). Tannic acid exhibits anti-inflammatory effects on formalin-induced paw edema model of inflammation in rats. Hum Exp Toxicol.

[28] Iwata N, Okazaki M, Kamiuchi S, Hibino Y (2010). Protective effects of oral administrated ascorbic acid against oxidative stress and neuronal damage after cerebral ischemia/reperfusion in diabetic rats. Journal of Health Science.

[29] Dixon WJ (1965). The up-and-down method for small samples. Journal of the American Statistical Association.

[30] Vissers K, Meert T (2005). A behavioral and pharmacological validation of the acetone spray test in gerbils with a chronic constriction injury. Anesth Analg.

[31] Owoyele BV, Bakare AO, Ayinla MT, Adeshina KA, Onietan D, Azeez SO (2021). Antinociceptive effects of lead acetate in sciatic nerve chronic constriction injury model of peripheral neuropathy in male Wistar rats. Naunyn Schmiedebergs Arch Pharmacol.

[32] Khan FA, Ali G, Rahman K, Khan Y, Ayaz M, Mosa OF (2022). Efficacy of 2-Hydroxyflavanone in rodent models of pain and inflammation: Involvement of opioidergic and GABAergic anti-nociceptive mechanisms. Molecules.

[33] Abolarin PO, Nafiu AB, Oyewole AL, Amin A, Ogundele OM, Owoyele BV (2021). Selenium reduces nociceptive response in acute 1-methyl-4-phenyl-1, 2, 3, 6-tetrahydropyridine (MPTP)-induced neurotoxicity. IBRO Neurosci Rep.

[34] Naples JG, Ruckenstein MJ, Singh J, Cox BC, Li D (2020). Intratympanic diltiazem-Chitosan hydrogel as an otoprotectant against cisplatin-Induced ototoxicity in a mouse model. Otol Neurotol.

[35] Hidayat R, Wulandari P (2021). Protocol for anesthesia animal model in biomedical study. Bioscientia Medicina: Journal of Biomedicine & Translational Research.

[36] Ohkawa H, Ohishi N, Yagi K (1979). Assay for lipid peroxides in animal tissues by thiobarbituric acid reaction. Anal Biochem.

[37] Condon ME, Petrillo Jr EW, Ryono DE, Reid JA, Neubeck R, Puar M (1982). Angiotensin-converting enzyme inhibitors: importance of the amide carbonyl of mercaptoacyl amino acids for hydrogen bonding to the enzyme. J Med Chem.

[38] Sun Y, Oberley LW, Li Y (1988). A simple method for clinical assay of superoxide dismutase. Clin Chem.

[39] Raeeszadeh M, Karimfar B, Amiri AA, Akbari A (2021). Protective effect of nano-vitamin C on infertility due to oxidative stress induced by lead and arsenic in male rats. Journal of Chemistry.

[40] Malik I, Shah FA, Ali T, Tan Z, Alattar A, Ullah N (2020). Potent natural antioxidant carveol attenuates MCAO-stress induced oxidative, neurodegeneration by regulating the Nrf-2 pathway. Front Neurosci.

[41] Bancroft JD, Gamble M (2008). Theory and practice of histological techniques.

[42] Patel S, Sangeeta S (2019). Pesticides as the drivers of neuropsychotic diseases, cancers, and teratogenicity among agroworkers as well as general public. Environ Sci Pollut Res Int.

[43] Kim KH, Kabir E, Jahan SA (2017). Exposure to pesticides and the associated human health effects. Sci Total Environ.

[44] Benbrook CM (2016). Trends in glyphosate herbicide use in the United States and globally. Environ Sci Eur.

[45] Baier CJ, Gallegos CE, Raisman-Vozari R, Minetti A (2017). Behavioral impairments following repeated intranasal glyphosatebased herbicide administration in mice. Neurotoxicol Teratol.

[46] Ait-Bali Y, Ba-M’hamed S, Gambarotta G, Sassoè-Pognetto M, Giustetto M, Bennis M (2020). Pre-and postnatal exposure to glyphosate-based herbicide causes behavioral and cognitive impairments in adult mice: evidence of cortical ad hippocampal dysfunction. Arch Toxicol.

[47] Li Y, Zhang C, Yin Y, Cui F, Cai J, Chen Z, Jin Y (2014). Neurological effects of pesticide use among farmers in China. Int J Environ Res Public Health.

[48] Davies KJ (2000). Oxidative stress, antioxidant defenses, and damage removal, repair, and replacement systems. IUBMB Life.

[49] Hacimuftuoglu A, Handy CR, Goettl VM, Lin CG, Dane S, Stephens Jr RL (2006). Antioxidants attenuate multiple phases of formalin-induced nociceptive response in mice. Behav Brain Res.

[50] Gallegos CE, Bartos M, Gumilar F, Raisman-Vozari R, Minetti A, Baier CJ (2020). Intranasal glyphosate-based herbicide administration alters the redox balance and the cholinergic system in the mouse brain. Neurotoxicology.

[51] Winstone JK, Pathak KV, Winslow W, Piras IS, White J, Sharma R (2022). Glyphosate infiltrates the brain and increases pro-inflammatory cytokine TNFα: implications for neurodegenerative disorders. J Neuroinflammation.

[52] Kummer KK, Mitrić M, Kalpachidou T, Kress M (2020). The medial prefrontal cortex as a central hub for mental comorbidities associated with chronic pain. Int J Mol Sci.

[53] Kumar J, Haldar C, Verma R (2021). Melatonin ameliorates LPS-induced testicular nitro-oxidative stress (iNOS/TNFα) and inflammation (NF-kB/COX-2) via modulation of SIRT-1. Reprod Sci.

[54] Dos Santos GG, Delay L, Yaksh TL, Corr M (2020). Neuraxial cytokines in pain states. Front Immunol.

[55] Levin SG, Pershina EV, Bugaev-Makarovskiy NA, Chernomorets IY, Konakov MV, Arkhipov VI (2021). Why do levels of antiinflammatory cytokines increase during memory acquisition?. Neuroscience.

[56] Kuffler DP (2020). Mechanisms for reducing neuropathic pain. Mol Neurobiol.

[57] Huang Y, Zhu L, Zhang W, Tang Q, Zhong Y (2022). IL-10 alleviates radicular pain by inhibiting TNF-α/p65 dependent Nav1.7 up-regulation in DRG neurons of rats. Brain Res.

[58] Jang Y, Kim M, Hwang SW (2020). Molecular mechanisms underlying the actions of arachidonic acid-derived prostaglandins on peripheral nociception. J Neuroinflammation.

[59] Campos ACP, Antunes GF, Matsumoto M, Pagano RL, Martinez RCR (2020). Neuroinflammation, pain and depression: An overview of the main findings. Front Psychol.

[60] Baptista-de-Souza D, Tavares LRR, Canto-de-Souza L, Nunes-de-Souza RL, Canto-de-Souza A (2022). Behavioral, hormonal, and neural alterations induced by social contagion for pain in mice. Neuropharmacology.

[61] Yu S, Li W, Shen W, Edwards RR, Gollub RL, Wilson G (2020). Impaired mesocorticolimbic connectivity underlies increased mechanical pain sensitivity in chronic low back pain. Neuroimage.

[62] Kaur A, Singh L, Garg S, Kaur H, Singh N, Bhatti R (2021). Involvement of oxidative stress and nerve growth factor in behavioral and biochemical deficits of experimentally induced musculoskeletal pain in mice: Ameliorative effects of heraclin. J Mol Neurosci.

[63] Neugebauer V (2002). Metabotropic glutamate receptors-important modulators of nociception and pain behavior. Pain.

[64] Zhang HM, Chen SR, Pan HL (2009). Effects of activation of group III metabotropic glutamate receptors on spinal synaptic transmission in a rat model of neuropathic pain. Neuroscience.

[65] Osikowicz M, Mika J, Przewlocka B (2013). The glutamatergic system as a target for neuropathic pain relief. Exp Physiol.

[66] Olmos G, Lladó J (2014). Tumor necrosis factor alpha: a link between neuroinflammation and excitotoxicity. Mediators Inflamm.

[67] Cattani D, de Liz Oliveira Cavalli VL, Rieg CEH, Domingues JT, Dal-Cim T, Tasca CI (2014). Mechanisms underlying the neurotoxicity induced by glyphosate-based herbicide in immature rat hippocampus: involvement of glutamate excitotoxicity. Toxicology.

[68] Guignet M, Dhakal K, Flannery BM, Hobson BA, Zolkowska D, Dhir A (2020). Persistent behavior deficits, neuroinflammation, and oxidative stress in a rat model of acute organophosphate intoxication. Neurobiol Dis.

